# Proteolytic processing of a precursor protein for a growth-promoting peptide by a subtilisin serine protease in Arabidopsis

**DOI:** 10.1111/j.1365-313X.2008.03598.x

**Published:** 2008-08-01

**Authors:** Renu Srivastava, Jian-Xiang Liu, Stephen H Howell

**Affiliations:** Plant Sciences Institute, Iowa State UniversityAmes, IA 50011, USA

**Keywords:** Arabidopsis, phytosulfokine, subtilase, peptide hormone, tissue culture, fluorogenic peptide

## Abstract

Phytosulfokines (PSKs) are secreted, sulfated peptide hormones derived from larger prepropeptide precursors. Proteolytic processing of one of the precursors, AtPSK4, was demonstrated by cleavage of a preproAtPSK4–myc transgene product to AtPSK4–myc. Cleavage of proAtPSK4 was induced by placing root explants in tissue culture. The processing of proAtPSK4 was dependent on AtSBT1.1, a subtilisin-like serine protease, encoded by one of 56 subtilase genes in Arabidopsis. The gene encoding AtSBT1.1 was up-regulated following the transfer of root explants to tissue culture, suggesting that activation of the proteolytic machinery that cleaves proAtPSK4 is dependent on AtSBT1.1 expression. We also demonstrated that a fluorogenic peptide representing the putative subtilase recognition site in proAtPSK4 is cleaved *in vitro* by affinity-purified AtSBT1.1. An alanine scan through the recognition site peptide indicated that AtSBT1.1 is fairly specific for the AtPSK4 precursor. Thus, this peptide growth factor, which promotes callus formation in culture, is proteolytically cleaved from its precursor by a specific plant subtilase encoded by a gene that is up-regulated during the process of transfering root explants to tissue culture.

## Introduction

Phytosulfokines (PSKs) are a class of plant peptides discovered through the study of growth factors that mediate density-dependent growth in cell culture (Matsubayashi and Sakagami, 2006). Matsubayashi and Sakagami (1996) isolated and identified growth factors from conditioned medium that promoted the growth at low density of asparagus mesophyll cells in tissue culture. They identified a sulfated pentapeptide [H-Tyr(SO_3_H)-Ile-Tyr(SO_3_H)-Thr-Gln-OH, abbreviated sYIsYTQ], named PSK-α, and a sulfated tetrapeptide [H-Tyr(SO_3_H)-Ile-Tyr(SO_3_H)-Thr-OH], named PSK-β, that were active in the asparagus cell system.

Six genes encoding PSKs (*AtPSK1–6*) have been identified in Arabidopsis. Each encodes a preproprotein precursor of approximately 80 residues, with the YIYTQ peptide near their C-termini (Matsubayashi and Sakagami, 2006). Matsubayashi *et al.* (2006) generated transgenic Arabidopsis plants (AtPSK4ox) over-expressing one of the AtPSKs, and found that root growth and callus formation were slightly enhanced in over-expression lines, but otherwise the seedlings were phenotypically indistinguishable from wild-type.

A PSK receptor was first identified in carrot (*Daucus carota*) as a leucine-rich repeat receptor kinase (LRR-RK) (Matsubayashi *et al.*, 2002). Sequence information from the carrot protein (DcPSKR1) was used to identify an ortholog in Arabidopsis, AtPSKR1 (At2g02220). Matsubayashi *et al.* (2006) described a mutant with a Ds insertion in *AtPSKR1* (*pskr1-1*), and found that callus derived from the mutant was less sensitive to the growth-promoting effects of PSK in culture. However, they observed little difference in overall plant growth between wild-type, *pskr1-1* and AtPSKR1ox, an over-expression line. The most prominent characteristic of *pskr1-1* was that vegetative tissues in mature plants lost their ability to form callus. Unlike wild-type plants, leaf discs from the fully expanded leaves of *pskr1-1* plants were less capable of producing callus, while unexpanded leaves retained callus-forming capacity. *AtPSKR1* over-expressing plants (AtPSKR1ox) showed delayed senescence, and, as a result, leaves continued to expand, resulting in larger leaves than the wild-type.

Little is known about the proteolytic processing of the PSK propeptide precursors, other than the fact that the precursors have conserved di-basic residues 8-10 amino acids upstream from the mature peptide sequence (Matsubayashi and Sakagami, 2006). Di-basic residues are characteristic of substrate sites for subtilases, subtilisin-like serine proteases (Barr, 1991); therefore, we wished to determine whether any of the proteases encoded by the 56 subtilase genes in the Arabidopsis genome (Rautengarten *et al.*, 2005) are responsible for cleavage of the AtPSK4 precursor. In doing so, we identified a subtilase, AtSBT1.1, that is required for cleavage of the fusion protein AtPSK4–myc. Cleavage of AtPSK4–myc is induced in root explants, suggesting that release of the peptide hormone from the precursor protein is controlled, in part, by the proteolytic processing machinery.

## Results

### *Proteolytic cleavage of the PSK4 precursor* in vivo

In studies of shoot regeneration in Arabidopsis tissue culture, we found that expression of a subtilase gene *AtSBT1.1* (At1g01900) correlated with conditions for efficient shoot regeneration (Lall *et al.*, 2004). It was not clear what the causal connection might be between efficient shoot regeneration and expression of a gene encoding a serine protease. Most subtilases are predicted to be secreted proteins (Rautengarten *et al.*, 2005), so we reasoned that AtSBT1.1 could be involved in processing of an extracellular growth factor or receptor related to shoot regeneration. Growth factors that might require the action of AtSBT1.1 include peptide hormones, such as AtPSKs. AtPSKs promote callus formation in tissue culture (Matsubayashi and Sakagami, 2006), and are synthesized as preproproteins with signal peptides that target them to the secretory pathway and with prosequences that are processed during maturation of the peptide hormone.

**Figure 1:**
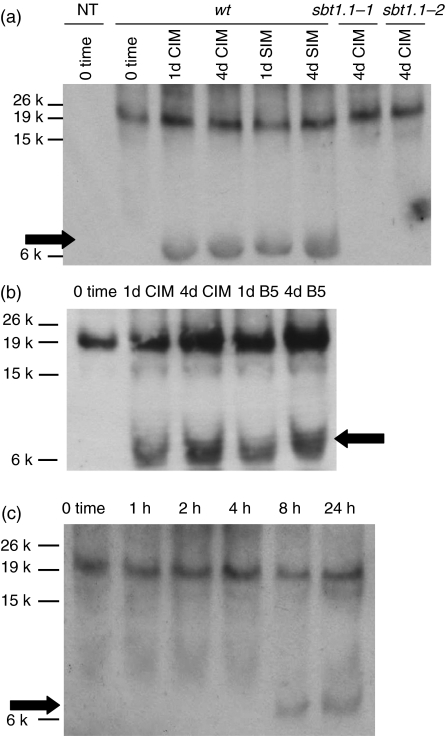
*In vivo* cleavage of pAtPSK4-myc. (a) Root segments from wild-type transgenic seedlings expressing the construct 35S:ppAtPSK4-myc were explanted and incubated on callus induction medium (CIM) for 1-4 days and then transferred onto shoot induction medium (SIM). Root segments from *sbt1.1-1* and *sbt1.1-2* mutants expressing 35S:ppAtPSK4-myc were also explanted and similarly incubated. Arrows indicate the predicted migration position for processed AtPSK4–myc. The lane marked NT is an extract from roots of non-transformed seedlings. (b) Root segments from seedlings bearing 35S:ppAtPSK4-myc in a wild-type background were explanted and incubated on normal CIM or on B5 basal medium, without cytokinin or auxin hormones. (c) Time course following explantation for acquisition of capacity to process pAtPSK4–myc on CIM medium.

To determine whether AtSBT1.1 is involved in the proteolytic processing of AtPSKs, we developed a constitutively expressed, C-terminal 4 x myc-tagged construct of the AtPSK4 precursor, 35S:ppAtPSK4–myc, and studied its processing *in vivo*. PreproAtPSK4 will be referred to as ppAtPSK4 and proAtPSK4 as pAtPSK4. AtPSK4 was chosen for study because it is the most abundantly expressed PSK precursor (Matsubayashi and Sakagami, 2006). The predicted size of pAtPSK4 with the myc tag is 12.8 kDa, but we observed a band at approximately 19 kDa on Western blots, larger than the predicted size (Figure 1a, wt, 0 time). To demonstrate, nonetheless, that this band is pAtPSK4–myc, we analyzed the partially purified myc-tagged protein and identified three peptides derived from the fusion protein by MS/MS analysis (Figure S1). Thus, the larger apparent size of pAtPSK4–myc may be due to anomalous gel migration behavior or post-translational modification of part of the precursor protein.

We were surprised to find that pAtPSK4–myc was not cleaved in roots of intact transgenic seedlings (Figure 1a, wt, 0 time). We suspected that failure to detect processing may have been due to seedling growth or culture conditions. We had chosen to study AtSBT1.1 in the first place due to observations that we had made about shoot regeneration from root explants (Lall *et al.*, 2004). Therefore, we subjected root explants from 35S:ppAtPSK4-myc seedlings to tissue culture conditions for regenerating shoots. This involves pre-incubating root segments on an auxin-rich callus induction medium (CIM), and then transferring them after 4 days to a cytokinin-rich shoot induction medium (SIM). Under these conditions, a band appeared at approximately 7 kDa in Western blots (Figure 1a, wt, lanes 1d and 4d CIM or SIM), representing the myc-tagged cleaved peptide, AtPSK4–myc. The size of the processed protein was consistent with a cut at or near the cleavage site as determined by the *in vitro* experiments described below. Thus, cleavage of pAtPSK4–myc appears to be induced by some aspect of the culturing process.

Having identified conditions for pAtPSK4–myc cleavage, we wished to determine whether AtSBT1.1 was responsible for the proteolysis. To do so, we examined cleavage of pAtPSK4–myc in T-DNA mutants with insertions in AtSBT1.1. The gene encoding AtSBT1.1 (At1g01900) has a single intron, and *sbt1.1-1* (SALK_111561) and *sbt1.1-2* (SALK_108704) have T-DNA insertions in the first exon. The T-DNA lines were judged to be null mutants because AtSBT1.1 transcripts were not found in seedlings from either line (Figure S2). The 35S:ppAtPSK4-myc construct was introduced into the two T-DNA mutant lines, and root explants were subjected to regeneration conditions (4 days incubation on CIM). The pAtPSK4–myc precursor was produced in these lines, but the cleavage product AtPSK4–myc peptide was not detected (Figure 1a, lanes *sbt1.1-1* and *sbt1.1-2*). To demonstrate that the transgenic product in these lines was still cleavable, we out-crossed *sbt1.1-1* bearing the 35S:ppAtPSK4-myc construct to wild-type, and demonstrated that processing was restored in root explants of the F_1_ seedlings (Figure S3). We concluded from these results that AtSBT1.1 is required for pAtPSK4–myc cleavage under these conditions. This finding is quite significant given that there are 56 different subtilases encoded by the Arabidopsis genome (Rautengarten *et al.*, 2005).

As shoot regeneration in culture is dependent on cytokinin and auxin hormones, we determined whether induction of pAtPSK4–myc cleavage required hormone treatment during CIM pre-incubation. To test this, root segments were explanted to hormone-free B5 medium as well as to CIM, and it was found that pAtPSK4–myc was cleaved on basal B5 medium at 1 and 4 days after explantation (Figure 1b). We concluded that the processing activity was probably induced by the wounding or handling of root tissue during explanting. We then determined how rapidly processing was induced. Cleavage of pAtPSK4–myc was first detected approximately 8 h after explanting (Figure 1c). Thus induction is fairly rapid, but not immediate as one might expect for a post-translational activation mechanism.

### *Up-regulation of* AtSBT1.1 *gene expression*

**Figure 2:**
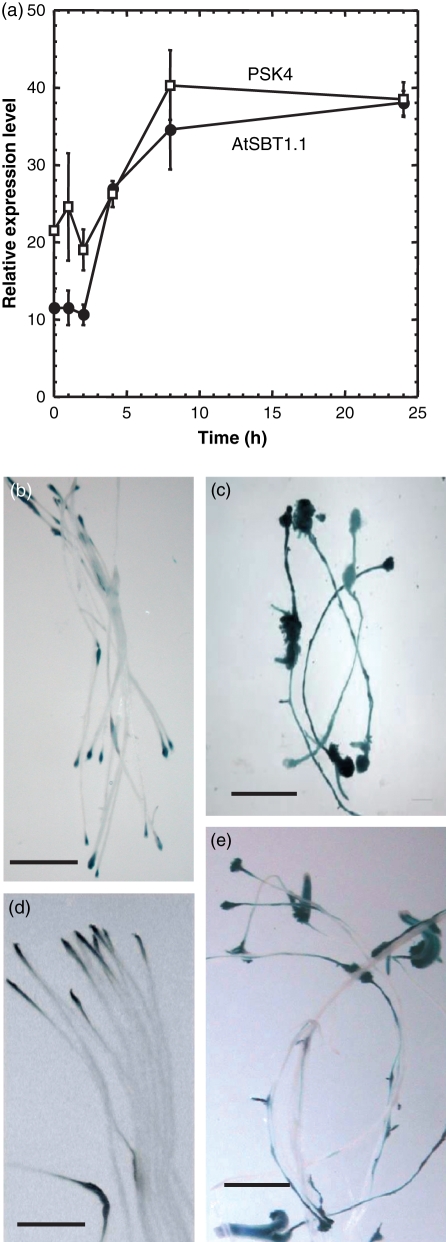
Expression profile of AtSBT1.1 and PSK4. (a) Quantitative RT-PCR was used to determine the abundance of AtSBT1.1 and PSK4 mRNA relative to actin probes. RNAs were extracted at the indicated times after explanting root segments on CIM. (b–e) Histochemical staining for GUS activity in root explants from transgenic plants expressing an AtPSK4 promoter–GUS fusion (b, c) or an AtSBT1.1 promoter–GUS fusion (d, e). Segments were stained after 4 days of incubation on CIM (b, d) or after 6 days of incubation on SIM (c, e). Scale bars = 1 mm.

As pAtPSK4 cleavage was induced approximately 8 h following the explanting of root segments, we wished to determine whether expression of the gene encoding AtSBT1.1 was similarly up-regulated. Real-time quantitative RT-PCR was performed on extracts from root segments at various times following explanting. It was found that AtSBT1.1 was up-regulated approximately 3.5-fold starting at approximately 8 h after explanting root segments (Figure 2a). Thus, the up-regulation of AtSBT1.1 expression was similar to the kinetics for the acquisition of cleavage activity. The endogenous gene encoding AtPSK4 was also up-regulated after explanting, but not as much as AtSBT1.1 (approximately twofold, Figure 2a).

We also developed promoter:GUS constructs for AtSBT1.1 and AtPSK4, and found that the gene was expressed at the cut ends of the root segments where callus formation first occurs during shoot regeneration (4 days CIM, Figure 2b,d). Later, AtSBT1.1 and AtPSK4 continued to be expressed most intensely at sites of callus and regenerative tissue formation, both at the ends and at other wound sites along the length of the root segments (6 days SIM, Figure 2c,e).

### Subcellular localization of AtSBT1.1 and AtPSK4

**Figure 3:**
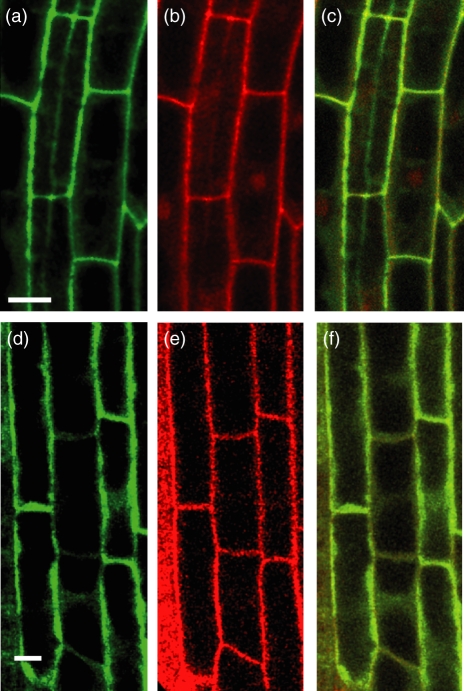
Subcellular localization of AtPSK4–YFP and AtSBT1.1–YFP. Confocal laser scanning microscope images of roots from seedlings expressing 35S:ppAtPSK4-YFP (a–c) or 35S:AtSBT1.1-YFP (d–f). (a, d) YFP fluorescence; (b, e) propidium iodide fluorescence; (c, f) merged images. Scale bars = 50 μm.

If pAtPSK4 is indeed a substrate for AtSBT1.1, then these proteins should occupy the same subcellular compartment. Both AtSBT1.1 and ppAtPSK4 have signal peptides, and both are predicted to be secreted proteins. To determine whether that is so, we constructed C-terminal YFP fusions and determined their location in root cells from Arabidopsis. Both AtPSK4–YFP (Figure 3a–c) and AtSBT1.1–YFP (Figure 3d–f) are associated with the periphery of the cell, coinciding with propidium iodide staining. Following plasmolysis (Von Groll *et al.*, 2002), most fluorescence from both YFP fusions remained associated with the extracellular matrix rather than the plasma membrane (Figure S4).

### *Cleavage of pPSK4* in vitro

To confirm that AtSBT1.1 is indeed involved in the processing of pAtPSK4–myc, we tested whether pAtPSK4 can be cleaved by AtSBT1.1 *in vitro*. To do so, we developed a pull-down assay using a C-terminal myc-tagged version of AtSBT1.1 (AtSBT1.1–myc) and a fluorogenic peptide substrate. We employed a similar assay in a previous study to test the activity of another Arabidopsis subtilase, AtS1P (Liu *et al.*, 2007a). In both cases, the tagged subtilases were synthesized in transgenic Arabidopsis because we were not able to produce active enzyme at high levels in heterologous systems.

**Figure 4:**
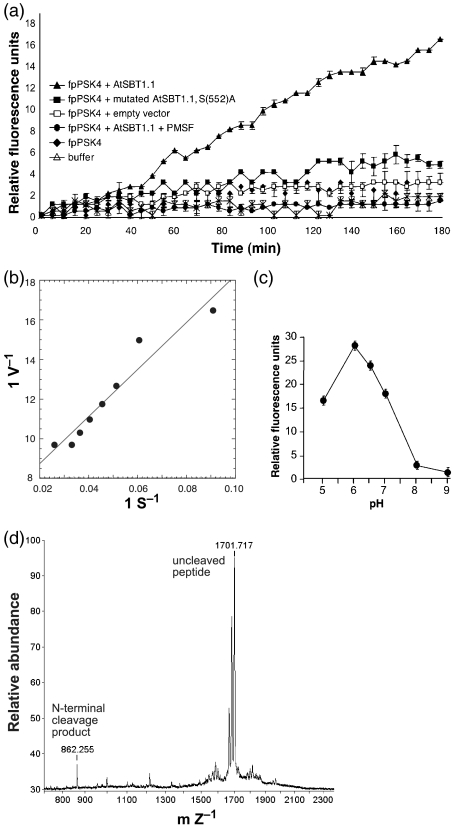
Cleavage reaction of the fluorogenic peptide representing the putative subtilase recognition site in pAtPSK4. (a) Time-dependent increase in fluorescence during incubation of the fluorogenic peptide fpAtPSK4 with bead-bound AtSBT1.1–myc. Various controls as indicated. (b) Lineweaver–Burk plot for AtSBT1.1–myc cleavage of fpAtPSK4. S is the concentration (μΜ), and V (reaction velocity) is in nmol min^−1^. Curve fit by linear regression (*R*^2^ = 0.929). *K*_m_ = 18.5 μm. (c) pH curve for cleavage of fpAtPSK4. The cleavage reaction was carried out for 90 min. (d) MALDI-TOF MS of cleavage of fpAtPSK4 by bead-bound AtSBT1.1. The MS spectrum was acquired after 30 min of reaction.

To test AtSBT1.1–myc for activity against pAtPSK4, we generated a fluorogenic peptide called fpAtPSK4 with the sequence Abz-RRSLVLHTDY(NO2)D-OH [where Abz is a 2-aminobenzoyl fluorescent group and Y(NO2)D-OH is a 3-nitrotyrosine quencher], representing a probable subtilase recognition site in pAtPSK4. The site was chosen because di-basic amino acid residues (in this case, RR) are characteristic signatures of subtilase recognition sites (Barr, 1991). When bead-bound protein from transgenic plants expressing AtSBT1.1–myc was incubated with fluorescence-quenched fpAtPSK4, a time-dependent increase in fluorescence was observed (Figure 4a). Because the enzyme was produced in a homologous system, we were mindful of potential contamination by endogenous Arabidopsis subtilases in our preparations. To preclude that possibility, we thoroughly washed the beads and routinely ran controls in which the same pull-down procedure was conducted with extracts from non-transgenic seedlings and from transgenic seedlings expressing a tagged subtilase (S552A) that was mutated at the active site (Figure 4a).

The kinetics of the reaction with the fluorogenic peptide substrate followed Michaelis–Menten kinetics, and the *K*_m_ for the reaction was determined to be approximately 18 μm (Figure 4b). The *K*_m_ value lies within the range of affinity constants for fluorogenic peptide substrates with comparable subtilases, such as mammalian prohormone convertases (Basak *et al.*, 2004,^2007^). The activity was inhibited by PMSF (Figure 4a), an inhibitor of serine proteases. The pH optimum for the reaction was in the acidic range, centered on pH 6 (Figure 4c).

To determine the site at which the peptide was cleaved, the reaction products were analyzed by MALDI-TOF analysis. The first N-terminal cleavage product that appeared had a molecular mass of 862.25, indicating a preferred cutting site at Abz-RRSLVL↓HTDY(NO2)D-OH (Figure 4d). MS/MS analysis of the 862.25 peak showed a spectrum of ions consistent with Abz-RRSLVL as the initial cleavage product (Figure S5). It should be noted that this cut is three amino acid residues upstream of the N-terminus of the mature peptide as has been described for PSKs in *Asparagus officinalis* (Matsubayashi and Sakagami, 1996). If the mature Arabidopsis peptide is similar, then further N-terminal proteolytic processing, as well as C-terminal processing, probably occurs to generate the active peptide.

### Substrate specificity of AtSBT1.1

**Figure 5:**
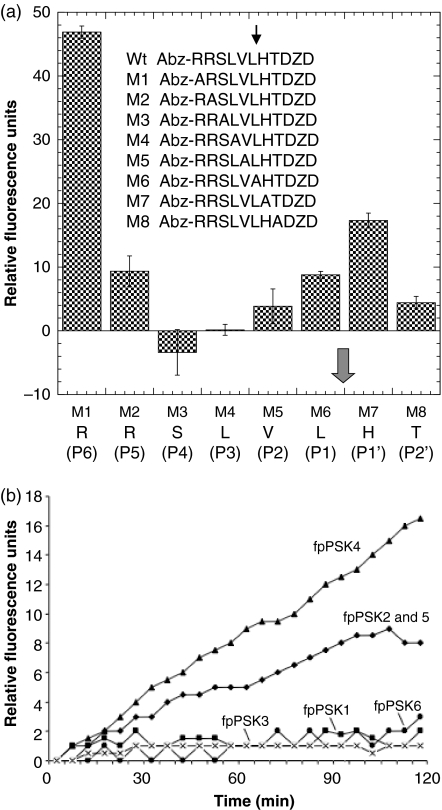
Specificity of AtSBT1.1 for pAtPSK4 as a substrate. (a) Alanine scan through the fluorogenic peptide, fpAtPSK4, representing the putative subtilase recognition site pAtPSK4. Alanines were substituted at the sites indicated in the various mutant versions of fpAtPSK4. The arrow shows the preferential cleavage site for bead-bound AtSBT1.1–myc. Fluorescence units are shown for 60 min of reaction with the various substrates. (b) Cleavage reactions catalyzed by bead-bound AtSBT1.1–myc for the fluorogenic peptides representing the putative subtilase recognition sites in the various AtPSK precursor proteins indicated in Table 1.

To determine whether AtSBT1.1 has specificity for the AtPSK4 recognition site, we conducted an alanine scan through fpAtPSK4, substituting one residue at a time for alanine (Figure 5a). The reactions were carried out in triplicate, and the reaction rates for each of the substitutions were compared to that for the wild-type substrate. The cleavage site sequence was very sensitive to alanine substitutions. Substituting the first arginine in the di-basic residues (P6 position) resulted in a reaction rate that was less than half that of wild-type. The most sensitive positions were P2–P4, which are modestly conserved among the AtPSK precursors.

**Table 1:** Amino acid sequences of Arabidopsis PSK proteins

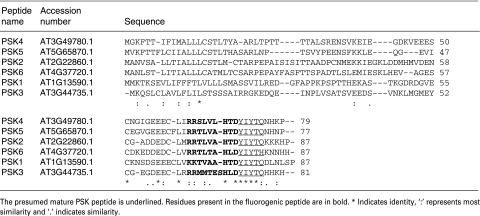

We compared the sequence of AtPSK4 to some of the most closely related AtPSKs and to PSK-related sequences in other plants. The five amino acids representing the presumed mature PSK peptide in the six Arabidopsis sequences are nearly identical (Table 1), as they are for other plant PSKs in the GenBank database, except for AtPSK6. The upstream di-basic amino acids and the leucine (P3 position) and histidine and aspartate (P1′ and P3′, respectively) are also conserved in Arabidopsis. To compare the activity of AtSBT1.1 with other AtPSK precursors, we developed fluorogenic peptides representing putative recognition sites for the family of AtPSKs (fpAtPSK1, 2, 3, 5 and 6, see bold residues in Table 1). The putative recognition sites for pAtPSK2 and 5 are the same and are represented in the fluorogenic peptides fpPSK2 and 5. The cleavage reaction was slower for fpAtPSK2 and 5 than for fpAtPSK4, and was barely detectable or not detectable at all with the other fpAtPSKs (Figure 5b). Thus, the activity of AtSBT1.1 is fairly specific, with most activity directed toward cleavage of pAtPSK4, followed by pAtPSK2 and 5.

## Discussion

The proteolytic processing of PSK4 appears to be highly regulated in the tissue culture system – in part by up-regulation of AtSBT1.1 gene expression. Among the 56 subtilases encoded in the Arabidopsis genome, AtSBT1.1 appears to be solely responsible for the release of PSK4 from its precursor in roots, because pAtPSK4–myc is not cleaved in AtSBT1.1 knock-out lines. PSKs are growth factors that stimulate callus formation in culture (Matsubayashi and Sakagami, 1996, 2006). Thus, up-regulation of AtSBT1.1 and the release of AtPSK4 from its precursor protein may be key factors in promoting the growth of cells and callus formation in tissue culture.

AtSBT1.1 functions similarly to prohormone convertases that release peptide hormones and neuropeptides from protein precursors in animal cells (Steiner, 1998). Prohormone convertases are subtilases in the secretory pathway that cleave substrates with mono- or di-basic amino acids in the general recognition motif R/K-Xn-R/K↓ (Seidah, 1997). In Arabidopsis, AtSBT1.1 cleaves substrates in a fairly site-specific manner, cleaving pAtPSK4 at RRSLVL↓HTDY and showing preference for cleavage of proAtPSK4 over other PSK precursors. Cleavage by AtSBT1.1 at the preferred cleavage site of the PSK4 precursor leaves three residues on the N-terminus of the five-residue peptide. This is of concern because Matsubayashi *et al.* (1996) demonstrated that even the presence of the tripeptide GGG severely reduces the activity of PSKs in an asparagus suspension cell system However, the three residues (HTD) remaining on the amino-end of AtSBT1.1-processed AtPSK4 are highly conserved among the PSKs, and it is possible that the sequence is part of the active, mature peptide or serves as a recognition signal for further processing by enzymes such as tripeptyl peptidases (Book *et al.*, 2005). Complete proteolytic processing of PSK4 probably involves a number of other steps, including trimming of the peptide at its C-terminus (Matsubayashi and Sakagami, 2006).

The preferred AtSBT1.1 cleavage site within the recognition motif for proAtPSK4 is somewhat unconventional, because the di-basic residues are usually in the P1 and P2 positions, immediately upstream (on the N-terminal side) of the cleavage site (Barr, 1991), The only caveat we have about the site is that it was identified from the cleavage product of a short fluorogenic peptide representing the putative subtilase recognition site. Other structural features of the intact AtPSK precursor may be important in determining the site of cleavage, but are not found in the peptide substrates that we used to characterize the cleavage reaction.

We have shown that YFP fusions of AtSBT1.1 and PSK4 accumulate in the extracellular matrix, and it is probable that cleavage occurs there simply because AtSBT1.1 has a slightly acid pH optimum and the apoplast is acidic (Bibikova *et al.*, 1998). PSK4 is tyrosine-sulfated (Matsubayashi and Sakagami, 1996), and the protein is likely to be sulfated in the *trans*-Golgi as are other sulfated proteins in animal cells (Baeuerle and Huttner, 1987). Therefore, it seems reasonable that the precursor is sulfated before it is cleaved. However, the precursor does not have to be sulfated to be cleaved, because AtSBT1.1–myc can cleave the unsulfated peptide, fpAtPSK4. Sulfation, however, is important for the function of the peptide, because the sulfated peptide binds to the PSK receptor (Matsubayashi *et al.*, 2002).

Although there are six genes encoding PSKs in Arabidopsis, the mature peptides (YIYTQ) encoded by each gene are identical (with the exception of AtPSK6, YIYTH). Presumably, the peptides encoded by each gene should be able to bind and activate the single known receptor, AtPSKR1 (Matsubayashi *et al.*, 2006). Each of the AtPSK precursors has the typical subtilase recognition site signature (di-basic amino acids) 8-10 residues upstream from the mature peptide (Barr, 1991). However, the residues that are critical for AtSBT1.1 recognition (the four or five residues just downstream from the di-basic site) differ somewhat between AtPSK genes, and therefore AtSBT1.1 appears to be most specific for cleavage of PSK4. That suggests that other subtilases might be involved in the processing of other PSKs.

Our interest in AtSBT1.1 stems from our earlier studies of shoot regeneration in Arabidopsis (Lall *et al.*, 2004). We found that higher expression levels of the gene encoding AtSBT1.1 (At1g01900) correlated with the presence of the superior allele at the major QTL conditioning shoot regeneration in Arabidopsis tissue culture (Lall *et al.*, 2004). Higher levels of AtSBT1.1 expression may not have anything to do directly with shoot regeneration. However, higher levels of AtSBT1.1 expression might promote the proliferation of callus from which shoots are derived. Similar reasoning was used by Hanai *et al.* (2000) to explain the stimulatory effects of PSKs on somatic embryo formation in carrot. They concluded that PSKs might promote the proliferation of cells giving rise to somatic embryos, rather than influencing the formation of somatic embryos.

## Experimental procedures

### Plant material and growth conditions

Two T-DNA insertion mutant lines for AtSBT1.1 were obtained from the Arabidopsis Biological Resource Center (ABRC, Columbus, OH). Seeds were surface-sterilized, rinsed with sterile water, and stratified at 4°C for at least 2 days in 0.1% agar. Seeds were germinated and grown vertically on agar plates containing Gamborg's B5 medium (Gamborg *et al.*, 1968). Root segments (5 mm) were cut and transferred to callus induction medium (CIM), which consisted of B5 medium with 5 g l^−1^ MES, 2.2 μm 2,4-dichlorophenoxyacetic acid, 0.2 μm kinetin and 0.8% agarose. Explants were incubated on CIM for 4 days under constant light conditions, and then transferred to shoot induction medium (SIM). SIM is prepared similarly to CIM except that it contains the hormones isopentenyladenine (5.0 μm) and 3-indoleacetic acid (0.9 μm). For AtSBT1.1–YFP and AtPSK4–YFP localization experiments, transgenic seedlings were grown on B5 plates for 7 days, and roots were used for confocal microscope examination. Screening for homozygous plants was carried out by PCR using left border (LB) T-DNA primers and the gene-specific primer pair ScrSBT1.1 (Table S1). The transcript level of AtSBT1.1 was evaluated by RT-PCR using primer pair sqRT-SBT1.1 listed in Table S1.

### Plasmid construction

ppAtPSK4 and AtSBT1.1 were amplified from root RNA of 1-week-old Arabidopsis seedlings by RT-PCR (primer pairs pSKMPSK4 and pSKMSBT1.1, respectively, Table S1), and cloned into the *Asc*I and *Spe*I sites of pSKM36 in-frame with a 4 x epitope myc tag (EQKLISEEDLRN). cDNA clones with error-free copies were named ppSKAtPSK4 and pSKAtSBT1.1, respectively. A mutated form of AtSBT1.1 (S552A) was generated using a QuickChange site-directed mutagenesis kit (Stratagene, http://www.stratagene.com/) with primer pairs SDM1.1 and pSKAtSBT1.1 as template. YFP C-terminal fusions were created by inserting cDNAs from above at the *Asc*I and *Spe*I sites of pSKY36. The clones were named 35S:AtSBT1.1-YFP and 35S:ppAtPSK4-YFP. Promoter–GUS constructs for AtSBT1.1 and AtPSK4 were generated by amplifying 984 and 934 nucleotides using the primers pCAMSBT1.1 and pCAMPSK4, respectively. The promoters were ligated into the *Bam*HI and *Pst*I sites of pCAMBIA3300.

### *Detection of AtSBT1.1 activity* in vivo

Total protein was extracted from transgenic and wild-type plants using extraction buffer [0.1 m HEPES/KOH pH 7.0, 20 mm 2-mercaptoethanol, 0.1 mg ml^−1^ PMSF, 0.1% w/v Triton X-100, 1 mm EDTA, 20% w/v glycerol and protease inhibitor cocktail (Sigma-Aldrich, http://www.sigmaaldrich.com/)]. An aliquot of total protein was precipitated using trichloroacetic acid and quantified by the Bradford method (Bradford, 1976). Reaction products were resolved by 12% SDS–PAGE and visualized by Western blotting using c-myc antibody (9E10; Santa Cruz Biotechnology, http://www.scbt.com) and an ECL kit (GE Healthcare, http://www.gehealthcare.com).

### *Assay for AtSBT1.1 activity* in vitro

AtSBT1.1–myc was affinity-purified from transgenic Arabidopsis plants as follows: 500 g of seedlings were ground in liquid nitrogen and suspended in 25 mm Tris/HCl pH 7.2, 150 mm NaCl, 0.1% Nonidet P-40 (Calbiochem, http://www.emdbiosciences.com) and 10% glycerol. Anti-c-myc agarose conjugate (200 μl; Sigma) was added to the filtered lysate and incubated for 2 h at 4°C with continuous rotation. The agarose beads with bound AtSBT1.1–myc were recovered by centrifugation at 1000 ***g*** for 3 min at 4°C. The beads were washed three times with washing buffer (25 mm Tris/HCl pH 7.2, 150 mm NaCl) and suspended in 25 mm MES/sodium acetate buffer pH 6.0. The reactions were carried out at 32°C in the same buffer supplemented with 2.5 mm CaCl_2_. Parallel purification was performed using transgenic plants transformed with a mutated form (S552A) of AtSBT1.1-myc and the empty vector to obtain material for control reactions.

For fluorogenic peptide assays, 40 μl of bead-bound AtSBT1.1–myc were added to a solution containing a final concentration of 50 μm fluorogenic peptide in a buffer consisting of 25 mm MES/sodium acetate pH 6.0 supplemented with 2.5 mm CaCl_2_. Kinetic assays were performed at 32°C and monitored as fluorescence emission at 420 nm (10 nm slit) following excitation by 320 nm (10 nm slit) in a BioTek spectrophotometer (http://www.biotek.com). The reaction was carried out in 96-well plates (Nunc, http://www.nuncbrand.com). Control reactions were performed using the same fluorogenic peptide with bead-bound mutated AtSBT1.1 (S552A) and bead-bound myc vector only. AtSBT1.1 incubated with 1 mm PMSF, peptide and reaction buffer, and buffer alone incubated in separate wells served as additional controls. To determine the pH optimum of the reaction, a tri-component buffer system of constant ionic strength was used (Ellis and Morrison, 1982). This buffer comprised 25 mm acetic acid, 25 mm MES, 50 mm Tris/HCl and 2.5 mm CaCl_2_.

### Gene expression analysis

Total RNA was isolated from ground plant tissues using an RNeasy kit (Qiagen, http://www.qiagen.com/), treated with RNase-free DNase I according to manufacturer's instructions (Qiagen), and quantified by 260/280 nm UV light absorption. A 1 μg aliquot of total RNA was reverse-transcribed using the Superscript III reverse transcription kit (Invitrogen, http://www.invitrogen.com/). Aliquots (2 μl) of cDNA were used for RT-PCR, and tenfold diluted cDNA was used for real-time quantitative RT-PCR. All primers are listed in Table S1. For real-time quantitative RT-PCR, the efficiency of amplification of various RNAs was assessed relative to amplification of transcripts for two actin genes [actin2 (At3g18780) and actin8 (At1g49240)]. RNA samples were assayed in triplicate. Expression levels were calculated relative to actin using a comparative threshold cycle method with ΔΔCt = ΔCt_reference_ − ΔCt_sample_, where ΔCt_sample_ is the Ct value for the assay sample normalized to actin and ΔCt_reference_ is the Ct value for calibration, also normalized to actin (Liu *et al.*, 2007b).

### GUS staining

Root segments (5 mm) incubated on CIM were harvested and further incubated for 6 h in GUS staining solution [100 mm Tris/NaCl buffer, pH 7, 2 mm ferricyanide, 2 mm X-gluc (5-bromo-4-chloro-3-indolyl-d-glucuronide), 2 mm ferrocyanide, 10 mm EDTA and 0.1% Triton X-100] at 37°C in the dark. The staining solution was removed, and the tissues were dehydrated in an ethanol series from 70% v/v to absolute ethanol. Samples were visualized under the light microscope.

### Mass spectrometry

MALDI-TOF MS and MS/MS analyses were performed using a QSTAR XL quadrupole TOF mass spectrometer (AB/MDS Sciex, http://www.appliedbiosystems.com) equipped with an orthogonal MALDI ion source. The mass spectrometer was operated in the positive ion mode. Mass spectra for MS analysis were acquired over *m*/*z* 500–2500. After every regular MS acquisition, MS/MS acquisition was performed against the most intensive ions. The molecular ions were selected by information-dependent acquisition in the quadrupole analyzer and fragmented in the collision cell.

### Confocal and epifluorescence imaging

Subcellular localization was carried out using 35S:pSKYAtSBT1.1-YFP- and 35S:pSKYAtPSK4-YFP-expressing roots. The roots were stained with propidium iodide and examined with a laser scanning confocal microscope. Confocal microscopy was performed using a Nikon C1si confocal scanning system attached to a 90i microscope (http://www.nikoninstruments.com). The emission signals for YFP and propidium iodide were acquired using sequential scanning mode to eliminate emission signal bleed-through. The 488 line of the argon laser and 515/30 emission filters were used for acquisition of YFP images. The propidium iodide images of root cells were acquired using the 561 argon laser and 590/50 emission filter.

Cells in root segments were plasmolyzed using the conditions described by Von Groll *et al.* (2002). Epifluorescent and differential interference contrast images of plasmolyzed cells were acquired using a 5 megapixel Nikon DS-Fi1 camera and Elements BR software. A 200 W mercury light source and FITC filter cube were used for image acquisition.

## Supporting Information

**Figure S1.** MS/MS analysis of pAtPSK4–myc.

**Figure S2.** RT-PCR analysis of AtSBT1.1 transcripts in wild-type and *sbt1.1-1* and *sbt1.1-2 mutants.*

**Figure S3.**pAtPSK4–myc processing is restored in F_1_ hybrids resulting from out-crossing of 35S:ppAtPSK4-myc *sbt1.1-1* to wildtype.

**Figure S4.** AtSBT1.1–YFP and AtPSK4–YFP are located in the apoplast.

**Figure S5.** MS/MS spectrum for the cleaved N-terminal fluorogenic peptide.

**Table S1.** Primer sequences used.
