# Supplementary material for: Proteolytic processing of a precursor protein for a growth-promoting peptide by a subtilisin serine protease in Arabidopsis
Source: Plant J. 2008 Aug 1;56(2):219–27. doi: 10.1111/j.1365-313X.2008.03598.x (PMC2667306; doi:10.1111/j.1365-313X.2008.03598.x)
Supplement: Supplementary file 1 [file tpj0056-0219-SD1.pdf]

(a)

MGKFTTIFIMALLLCSTLTYAARLTPTTTTALSRENSVKEIEGDKVEE

Signal peptide sequence 576.3 690.4

ESCNGIGEEECLIRRSLVLHTDYIYTQNHKPTSEQKLISEEDLRNEQK

974.5

LISEEDLRNEQKLISEEDLRNEQKLISEEDLR

974.5 974.5 974.5

Figure S1a

(b)

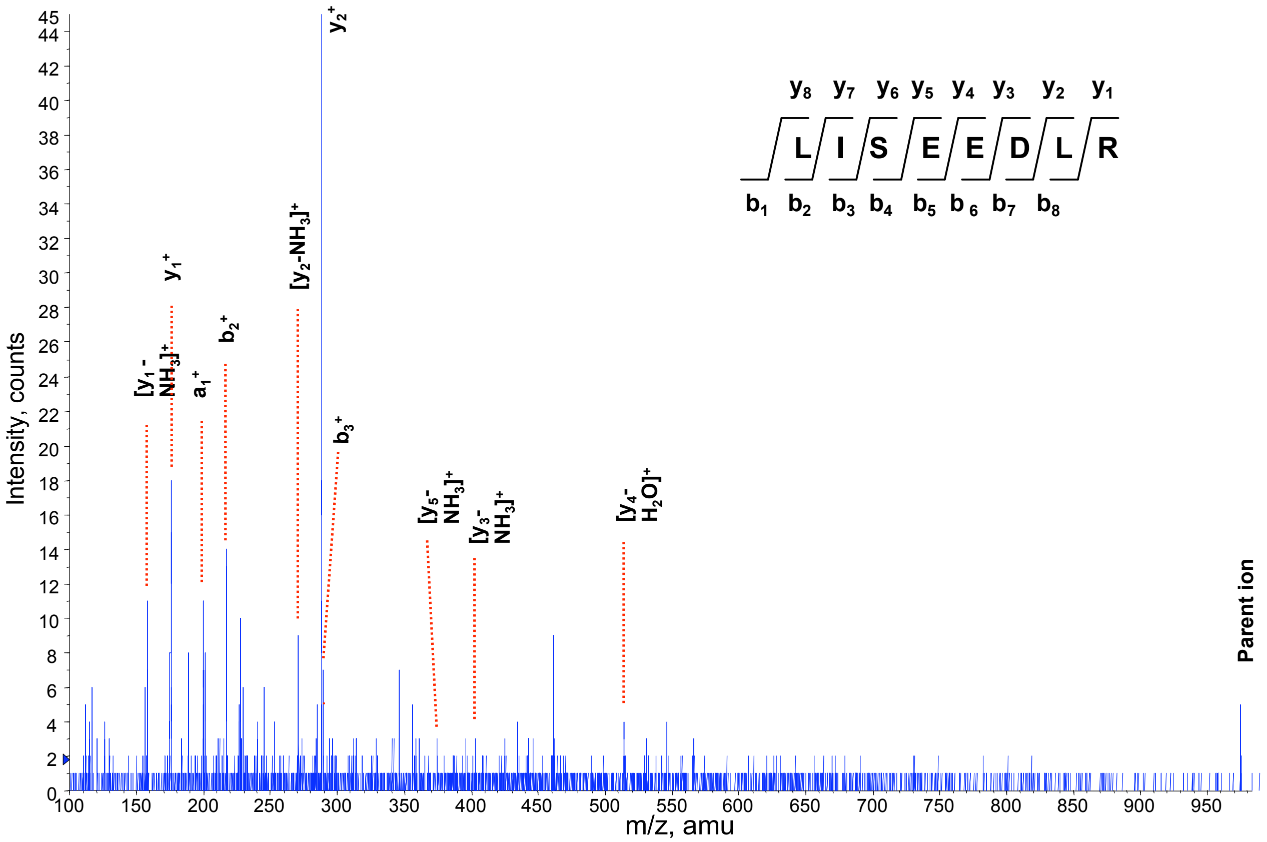

y8 y7 y6 y5 y4 y3 y2 y1
L I S E E D L R
b1 b2 b3 b4 b5 b 6 b7 b8
y2+
[y2-NH3]+
y1+
b2+
[y1 - NH3]+
a1+
b3+
[y5- NH3]+
[y3- NH3]+
[y4- H2O]+
Parent ion
Intensity, counts
m/z, amu

Figure S1b

(c)

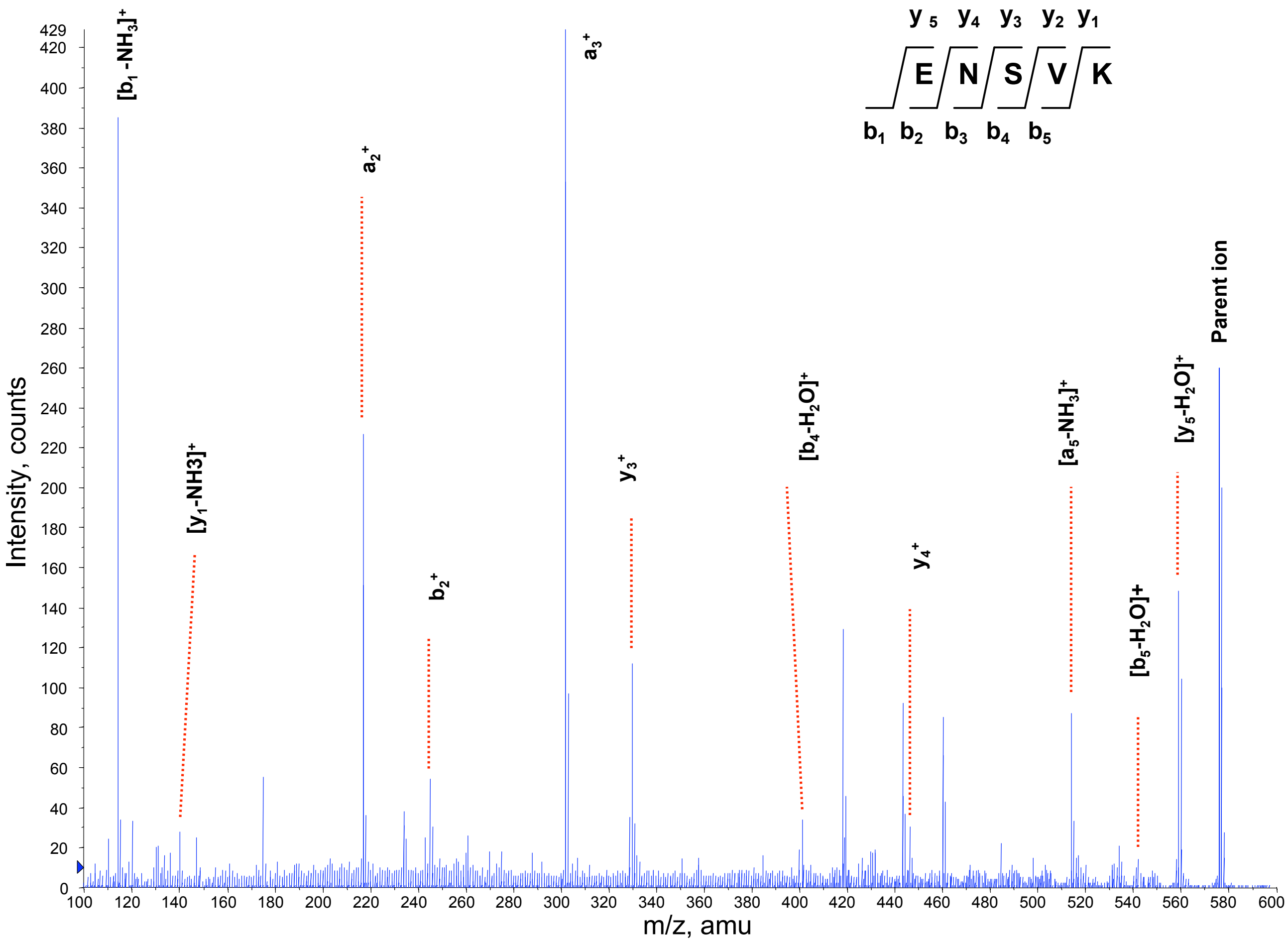
y 5 y4 y3 y2 y1
E N S V K
b1 b2 b3 b4 b5
[b1 -NH3]+
[y1-NH3]+
a2+
b2+
a3+
y3+
[b4-H2O]+
y4+
[a5-NH3]+
[b5-H2O]+
[y5-H2O]+
Parent ion
Intensity, counts
m/z, amu

Figure S1c

(d)

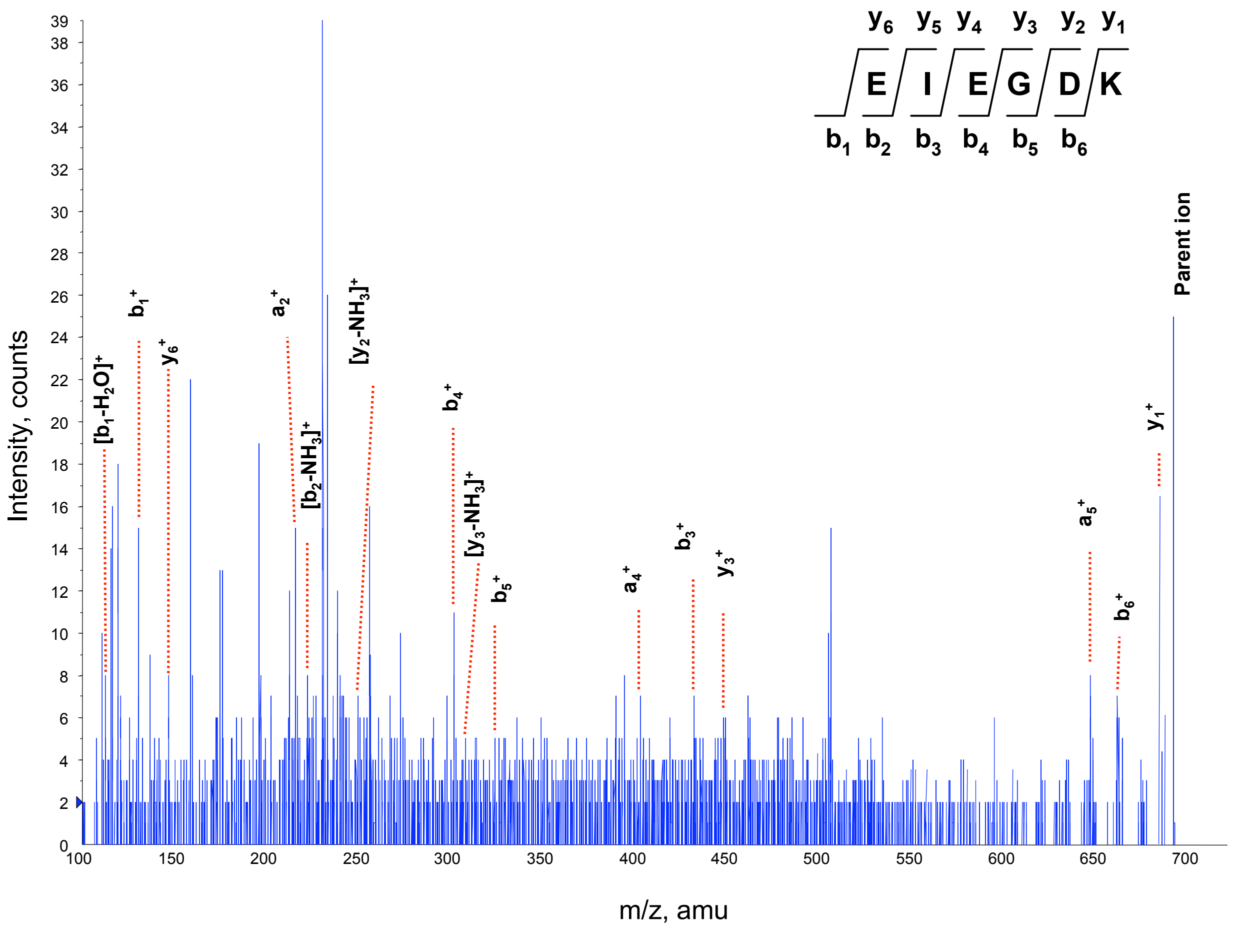

y6 y5 y4 y3 y2 y1
E I E G D K
b1 b2 b3 b4 b5 b6
[b1-H2O]+
b1+
y6+
a2+
[b2-NH3]+
[y2-NH3]+
b4+
[y3-NH3]+
b5+
a4+
b3+
y3+
a5+
b6+
y1+
Parent ion
Intensity, counts
m/z, amu

Figure S1d

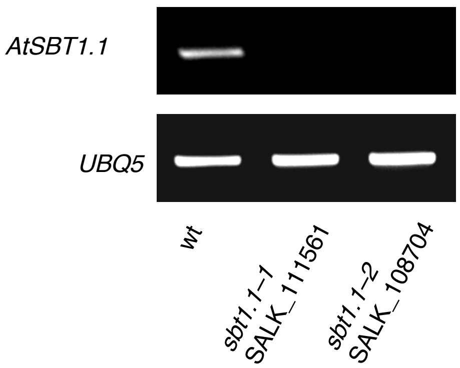

Figure S2

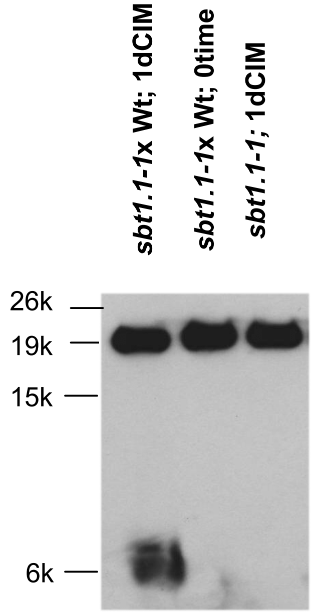
*sbt1.1-1*x Wt; 1dCIM
*sbt1.1-1*x Wt; 0time
sbt1.1-1; 1dCIM
26k
19k
15k
6k

Figure S3

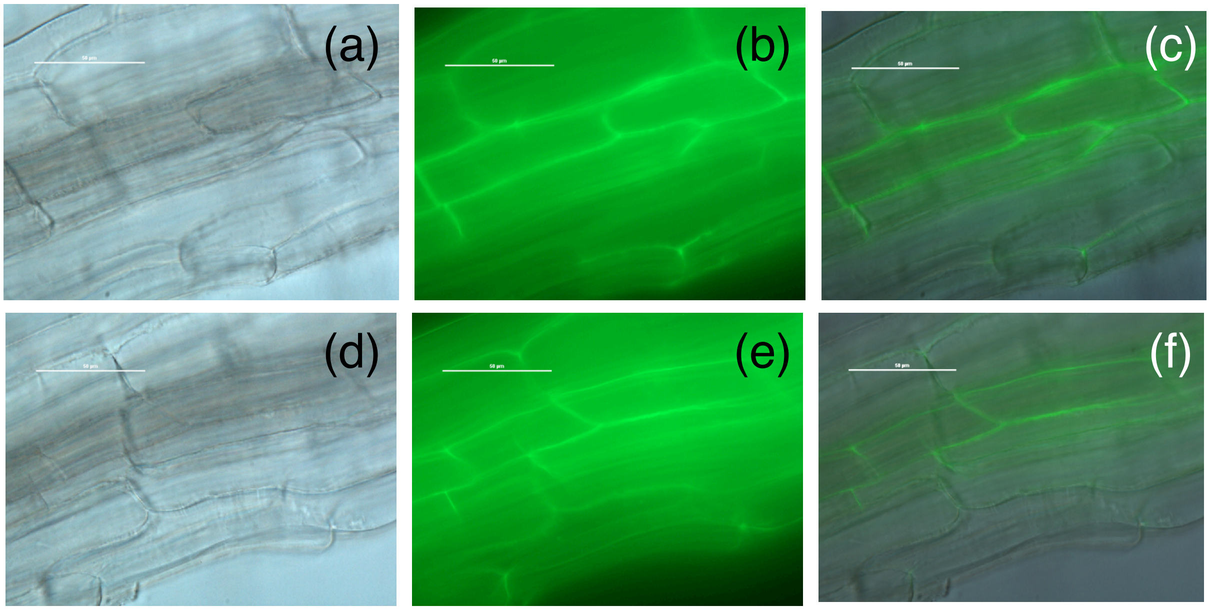

Figure S4

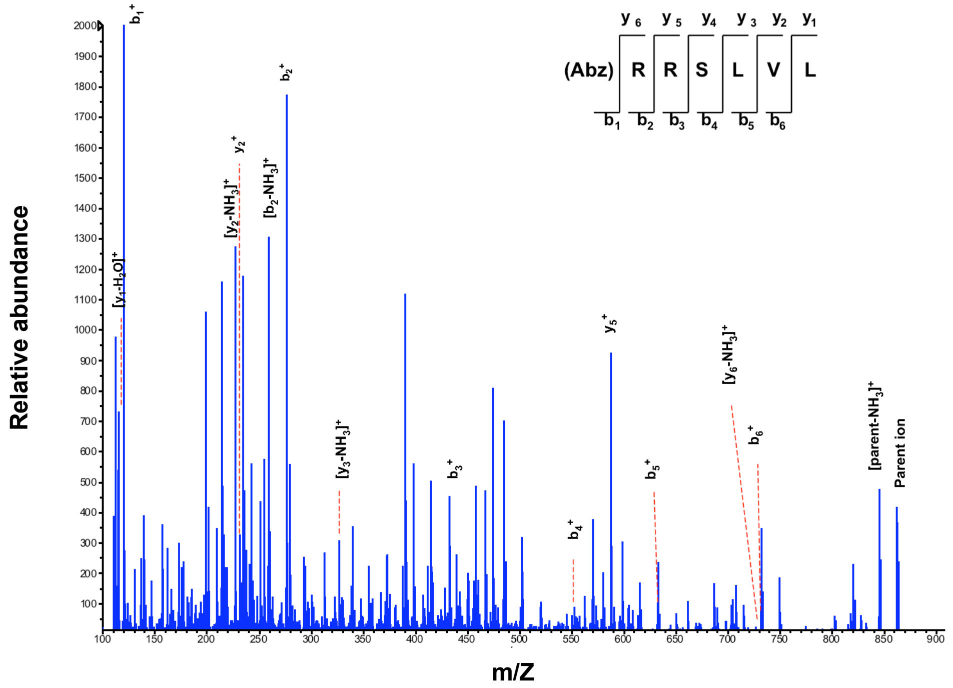

(Abz) R R S L V L
y6 y5 y4 y3 y2 y1
b1 b2 b3 b4 b5 b6
Relative abundance
m/Z

Figure S5
