# Supplementary material for: Proteolytic processing of a precursor protein for a growth-promoting peptide by a subtilisin serine protease in Arabidopsis
Source: Plant J. 2008 Aug 1;56(2):219–27. doi: 10.1111/j.1365-313X.2008.03598.x (PMC2667306; doi:10.1111/j.1365-313X.2008.03598.x)
Supplement: Supplementary file 2 [file tpj0056-0219-SD2.doc]

Supplemental table 1: Primers used for PCR and qRT-PCR analysis.

| **name** | **purpose** | **Forward primer** | **Reverse primer** |
| --- | --- | --- | --- |
| pSKMPSK4 | gene cloning | GAAGGCGCGCCATGGGTAAGTTCACAACCAT | GGACTAGTGGGCTTGTGATTCTGAGTAT |
| pSKYPSK4 | gene cloning | GAAGGCGCGCCATGGGTAAGTTCACAACCAT | GGACTAGTGGGCTTGTGATTCTGAGTAT |
| pSKMSBT1.1 | gene cloning | GAAGGCGCGCCATGCATCGCTTTCTTTTAATGCTCT | GGACTAGTTTCCCACGTCACGGCGATTGGGCTT |
| pSKYSBT1.1 | gene cloning | GAAGGCGCGCCATGCATCGCTTTCTTTTAATGCTCT | GGACTAGTTTCCCACGTCACGGCGATTGGGCTT |
| pCAMSBT1.1 | gene cloning | AACTGCAGTTTGTTGGTTAAGTATTACTCTATTCA | CGGGATCCCTACATGTGATTCTTAGGACTGGTCTC |
| pCAMPSK4 | gene cloning | AACTGCAGTCGATAGATCGGTGGTACCTTAC | CGCGGATCCACTGATTTGCAAAATAGAGAAAG |
| SDM1.1 | mutagenesis | CAATATCATCTCGGGAACCGCCATGGCTTGTCCTCACATC | GATGTGAGGACAAGCCATGGCGGTTCCCGAGATGATATTG |
| qRTSBT1.1 | qRT-PCR | TGTAGATGCATCGCTTTC | AAGAGCTCGTCTGGATAAGCA |
| qRTPSK4 | qRT-PCR | ATGGGTAAGTTCACAACCAT | GGGCTTGTGATTCTGAGTAT |
| qRTACT | qRT-PCR | GGTAACATTGTGCTCAGTGGTGG | AACGACCTTAATCTTCATGCTGC |
| sqRTSBT1.1 | sqRT-PCR | AGAATCACATGTAGATGCATCG | CTCGAGTCGACCTGAAATCAGT |
| sqRTUBQ5 | sqRT-PCR | TTGAAGACGGCCGTACCCTC | CGCTGAACCTTTCAAGATCCATCG |
| ScrSBT1.1 | genotyping | TGCACCTCATAAGACTAGACTTGTT | AACCTCATCCCTGAAGCTAAACCTT |
